# Moderate Associations Between the Use of Levonorgestrel-releasing Intrauterine Device and Metabolomics Profile

**DOI:** 10.1210/clinem/dgae318

**Published:** 2024-05-08

**Authors:** Elena Toffol, Oskari Heikinheimo, Pekka Jousilahti, Lara Lehtoranta, Anni Joensuu, Timo Partonen, Iris Erlund, Jari Haukka

**Affiliations:** Department of Public Health, Clinicum, Faculty of Medicine, University of Helsinki, 00014 Helsinki, Finland; Department of Obstetrics and Gynecology, University of Helsinki and Helsinki University Hospital, 00014 Helsinki, Finland; Department of Public Health and Welfare, Finnish Institute for Health and Welfare, FI-00271 Helsinki, Finland; Department of Public Health and Welfare, Finnish Institute for Health and Welfare, FI-00271 Helsinki, Finland; Department of Knowledge Brokers, Finnish Institute for Health and Welfare, FI-00271 Helsinki, Finland; Department of Public Health and Welfare, Finnish Institute for Health and Welfare, FI-00271 Helsinki, Finland; Department of Government Services, Finnish Institute for Health and Welfare, FI-00271 Helsinki, Finland; Department of Public Health, Clinicum, Faculty of Medicine, University of Helsinki, 00014 Helsinki, Finland

**Keywords:** metabolites, levonorgestrel-releasing intrauterine device, fertile-aged, metabolic changes, longitudinal, cardiometabolic risk

## Abstract

**Context:**

Use of a levonorgestrel-releasing intrauterine device (LNG-IUD) has become common irrespective of age and parity. To date, only a few studies have examined its possible metabolic changes and large-scale biomarker profiles in detail and in a longitudinal design.

**Objective:**

To apply the metabolomics technique to examine the metabolic profile associated with the use of LNG-IUD both in a cross-sectional and a longitudinal design.

**Design:**

The study consists of cross-sectional and longitudinal analyses of a population-based survey (Health 2000) and its 11-year follow-up (Health 2011). All participants aged 18 to 49 years with available information on hormonal contraceptive use and metabolomics data (n = 1767) were included. Altogether 212 metabolic measures in LNG-IUD users (n = 341) were compared to those in non-users of hormonal contraception (n = 1426) via multivariable linear regression models. Participants with complete longitudinal information (n = 240) were divided into continuers, stoppers, starters, and never-user groups, and 11-year changes in levels of each metabolite were compared.

**Results:**

After adjustment for covariates, levels of 102 metabolites differed in LNG-IUD current users compared to non-users of hormonal contraception (median difference in biomarker concentration: –0.12 SD): lower levels of fatty acids concentrations and ratios, cholesterol, triglycerides and other lipids, as well as particle concentration, cholesterol, total lipids, and phospholipids in lipoproteins. The 11-year metabolic changes did not differ in relation to changes in LNG-IUD use.

**Conclusion:**

The use of LNG-IUD was associated with several moderate metabolic changes, mostly suggestive of a reduced arterial cardiometabolic risk. Changes in LNG-IUD use were not related to long-term metabolic changes.

The use of hormonal intrauterine device (IUD), namely the levonorgestrel-releasing 52 mg IUD (LNG-IUD) and, more recently, its low-dose forms (13.5 mg and 19.5 mg LNG-IUDs), has rapidly increased worldwide since its first introduction in the Nordic countries in the early 1990s. While originally prescribed mainly to late reproductive age parous women, its use has become more common among all women irrespective of age and parity (1, 2). After insertion, the 52 mg LNG-IUD can currently be used for up to 7 to 8 years, and many users continue with subsequent device(s) well into menopause as part of menopausal hormonal therapy.

As an IUD, the mechanism of LNG-IUD action is mainly related to local effects on cervical mucus and on endometrium (3). Still, small concentrations of levonorgestrel pass into the systemic blood circulation, and systemic effects have been reported (4, 5). Thus, given the increasing number of women who opt for the LNG-IUD already at a young age and continue using it for several years, its possible long-term metabolic effects are of great clinical interest.

While the use of estrogen-containing combined contraceptives is a well-known independent risk factor for acute cardiovascular events such as increased risk of thrombosis, progestin-only contraceptives have a safer metabolic profile (6-8). For example, the metabolic effects of progestin-only contraceptive implants have been found to be null or minimal (9), and, according to several studies, the use of LNG-IUDs is not associated with increased risk for cardiovascular accidents (6, 10-13).

However, understanding the link between the use of hormonal contraception (HC) and possible long-term adverse cardiometabolic outcomes is especially challenging, given that such events are relatively rare among women of reproductive age, while they are more prevalent in the older age group. Nonetheless, metabolic perturbations directly related to the use of HC may stem already long before the index clinical event. Additionally, as use of subsequent LNG-IUDs is becoming more common, it is important to examine the potential effects of cumulative exposure at a young age on the long-term metabolic risks. For these reasons, surrogate measures, such as metabolic biomarkers, may be relevant early indicators of (increased or reduced) long-term risk. This information adds not only in terms of mechanistic knowledge but also in terms of preventive and clinical insights.

To date, only a few studies have examined in detail the possible metabolic changes and large-scale biomarker profiles associated with the use of LNG-IUDs using the metabolomics technique (14, 15). In the study by Wang et al (14), data from almost 500 users of the 52 mg LNG-IUD from 3 Finnish surveys were analyzed; only a few weak associations with any metabolic biomarkers were found in the cross-sectional part of the study and no associations in the longitudinal one. More recently, we examined 211 metabolites in more than 5000 late reproductive age users of LNG-IUD and non-users of HC between 1997 and 2017 in Finland and found several, mostly moderate perturbations, possibly suggestive of a protective metabolic profile (15). The aim of the current study was to replicate our previous findings, while adding longitudinal evidence, by analyzing data from a large population-based study carried out in Finland in 2000 and its 11-year follow-up study.

## Materials and Methods

Material for this study was selected from 2 population-based surveys conducted in Finland, namely the Health 2000 and the Health 2011 studies (16, 17). The Health 2000 was a nationally representative comprehensive survey consisting of interviews, questionnaires, and extensive health examinations conducted across 80 regions in Finland in 2000-2001; overall, more than 8000 people aged 30 years and over were invited to participate. The Health 2011 was a follow-up study conducted in 2011-2012. Over 8000 individuals who had participated in the Health 2000 survey and an additional sample of nearly 2000 young adults (18-29 years) were invited to participate in the follow-up study by means of health examination (inclusive of blood sampling), interviews, and/or questionnaires. The surveys were approved by the Coordinating Ethics Committee of the Helsinki and Uusimaa Hospital District (HUS Regional Committee on Medical Research Ethics). All participants gave written informed consent.

### Study Population

The population for this study included all fertile-aged (18-49 years) respondents with available (self-reported) information on their current and past use of HC and metabolomics data obtained from serum samples drawn in connection with the health examination.

In each survey, the following exclusion criteria were applied: current pregnancy, menopause (“Did your periods end?”, with answers “naturally with the menopause” or “because of an operation or radiotherapy”), current use of menopausal hormonal therapy, hysterectomy, and current use of oral contraceptive pills (or of vaginal ring or transdermal patch, information available only in Health 2011), resulting in a final population of 1767 individuals with available metabolomics data (341 currently using a LNG-IUD and 1426 HC non-users). Detailed sample size and background information for each survey are reported in Supplementary Table S1 (18) and Supplementary Fig. S1 (18).

Of this population, 240 of the women with metabolomics data who were using LNG-IUD or no HC in 2000 had also information on their LNG-IUD use and metabolomics data available at the Health 2011 and thus contributed to the sample used for the longitudinal study. Women who were using oral contraceptives in 2000 or 2011 were excluded from this part of the study.

### HC

Information on current and previous use of HC (pills and LNG-IUD) and its duration was obtained through questions during the health interview. All surveys additionally inquired into current use of nonhormonal IUDs; the Health 2011 also included information on the use of the contraceptive vaginal ring, patch, or other HC. The 52 mg LNG-IUD was the only hormonal IUD commercially available in Finland until 2013, with an approved maximum duration of use in Finland at the time of 5 years.

### Covariates

In addition to age, covariates obtained from the health examination, self-administered questionnaires, and clinical measurements included the following: season of sampling; alcohol use (yes, no/quit); frequency of smoking (every day, sometimes, never); physical activity (regular physical activity vs no regular physical activity); current use (during the past 7 days) of prescription drugs as listed in Table 1; chronic/severe diseases (heart diseases, hypertension, venous thrombosis, diabetes, psychological or mental illnesses, rheumatoid arthritis, osteoarthritis, cancer); and body mass index (BMI) based on the measured height and weight.

**Table 1.** Background characteristics of the study population (Pooled Health 2000 and Health 2011 data, n = 1767)

Characteristics	LNG-IUD users n = 341	LNG-IUD non-users n = 1426	*P*-value
Age*^a^*	41.5 (6.1)	39.5 (5.1)	<.0001
Age group (%)			<.0001
18-29 years	2 (0.6)	45 (3.2)	
30-39 years	111 (32.6)	637 (44.7)	
40-49 years	228 (66.9)	744 (52.2)	
Occupational status (%)			.0005
Full-time employment	271 (79.5)	980 (68.7)	
Part-time employment	29 (8.5)	106 (7.4)	
Student	6 (1.8)	59 (4.1)	
Retired	4 (1.2)	27 (1.9)	
Unemployed or temporarily laid-off	12 (3.5)	127 (8.9)	
Management of own household or care of family members	17 (5.0)	120 (8.4)	
Other	2 (0.6)	7 (0.5)	
BMI kg/m^2^	25.6 (4.7)	25.4 (5.1)	.58
Season of sampling (%)			.20
December-February	65 (19.1)	273 (19.2)	
March-August	25 (7.3)	70 (4.9)	
September-November	251 (73.6)	1083 (76.0)	
Alcohol use (%)			.0006
Yes	326 (96.2)	1275 (90.1)	
No/quit	13 (3.8)	140 (9.9)	
Smoking (%)			.76
Every day	73 (21.4)	320 (22.5)	
Sometimes	17 (5.0)	82 (5.8)	
No	251 (73.6)	1023 (71.8)	
Physical activity (%)			.33
Hardly any regular weekly physical activity	207 (75.0)	907 (71.9)	
Physical activity yes	69 (25.0)	355 (28.1)	
Parity (%)			<.0001
0	7 (2.1)	177 (13.9)	
1-3	306 (90.5)	994 (78.0)	
4 or more	25 (7.4)	104 (8.2)	
Current use (past week) of prescription drugs*^b^*	132 (39.4)	540 (39.5)	1
Chronic/severe disease*^c^*	117 (34.4)	540 (38.6)	.18

Abbreviations: BMI, body mass index; LNG-IUD, levonorgestrel-releasing intrauterine device.

^*a*^Range: 30-49 years in Health 2000; 18-49 in Health 2011.

^*b*^Insulin, oral hypoglycemics, antithrombotic therapy, lipid medications, combination products of estrogens and progestins, estrogens, progestogens, systemic corticosteroids, systemic antibiotics, systemic antimycotics, cytostatics, anti-inflammatory analgesics, opioids, other analgesics (no nonsteroidal anti-inflammatory drug group), migraine medications, epilepsy medications, antipsychotics, anxiolytics, hypnotics and sedatives, antidepressants, psycholeptics and psychoanaleptics in combination, long- and short-acting beta agonists, combination product of beta agonist and corticosteroids, inhaled corticosteroids, anticholinergics.

^*c*^Heart diseases, hypertension, venous thrombosis, diabetes, psychological or mental illnesses, rheumatoid arthritis, osteoarthritis, cancer.

### Metabolomics Measures

In each survey, the metabolomics measures were obtained from blood samples drawn in connection with the health examination, after fasting and without drinking on the same day, and after a minimum of 4-hour fasting. The serum and plasma samples were centrifuged at 1600 to 1800 g for 10 minutes and immediately frozen to −20 °C on site, normally within 45 to 60 minutes but no later than 90 minutes from sampling; thereafter, they were transferred to their final storage location (−70 °C), no later than 1 to 2 weeks after sampling. Serum sections were analyzed with a high-throughput serum nuclear magnetic resonance metabolomics platform (^1^H NMR Spectroscopy, Nightingale Health 2018-2019). Biomarkers were quantified independently for each serum sample. Nightingale's biomarker analysis technology applies a single experimental setup, and spectral information is converted to absolute concentrations (in molar units) of the metabolic measures. The platform allows for the simultaneous quantification of 250 metabolic biomarkers per sample, including 12 lipid measures of 14 lipoprotein subclasses [6 very-low-density lipoproteins, 4 high-density lipoproteins (HDLs), 3 low-density lipoproteins (LDLs), intermediate-density lipoprotein], other detailed molecular information on serum lipids (eg, sphingomyelin, fatty acids, etc.), or low molecular weight metabolites (eg, amino acids). An additional set of metabolite ratios, recognized as proxies for enzymatic activity and/or a biologically separate entity of measures, is also computed to obtain further clinical insight (19-21).

### Statistical Analyses

The study consists of a cross-sectional and a longitudinal part. After preliminary inspections of the metabolomics data in each cohort, metabolites with more than 100 missing observations were excluded from the analyses, resulting in a total of 212 metabolic measures; metabolic measures with value “zero” were replaced with 0.25 × the minimum observed value for that metabolite, and, after log transformation, remaining missing data were imputed through random forest imputation (22). Given the rather long time interval between the 2 surveys, in the cross-sectional part of the study, single observations were treated as independent, and the 2 datasets were pooled together on common variables. Analyses of associations were carried out via linear regression models, with each metabolic measure as the outcome variable and use of a hormonal IUD (vs current non-use of any HC) as the predictor of interest using the “ggforestplot” R-package (23). To exclude dependency issues related to some women providing data to both Health 2000 and Health 2011 surveys, analyses were repeated with generalized estimating equation (GEE) models (with the “gee” R-package, with exchangeable correlation structure). Three models were fitted: model 1, controlled for age, BMI, season of sampling, and study cohort; model 2, which is model 1 further controlled for diseases and medication use, which have a potential impact on both the choice of HC and the metabolic status; and model 3, which is model 1 further adjusted for lifestyle factors that likely affect metabolic status and choice of HC use, such as alcohol use, smoking, and physical activity. Direct acyclic graphs illustrating covariate selection for each model are reported in Supplementary Fig. S2 (18).

Regression model 3 was additionally repeated in age-stratified groups (18-39 years vs 40-49 years). As mentioned, metabolomics data of women younger than 30 years were available only in the Health 2011 survey; in addition, at the time of data collection, LNG-IUD was prescribed mostly to older and parous reproductive-age women. For these reasons, our overall sample of LNG-IUD users was underrepresented in the young age group. Hence, analyses were also repeated after exclusion of women younger than 30 years.

In order to allow the comparison across multiple measures, association magnitudes are reported in SD units of difference in biomarker concentration compared to the reference group. Results of analyses carried out separately in each of the 2 surveys (ie, Health 2000 and Health 2011) are reported as supplementary material (Supplementary Figs. S3-S5) (18).

Moreover, to examine the effect of duration of use on the metabolic profiles of hormonal IUDs, linear regression analyses (model 3) were repeated in the group of current users only, comparing intermediate-term (2 to 5 years) and long-term (more than 5 years) use of LNG-IUDs to short-term cumulative use (up to 2 years).

In order to test if the possible associations of LNG-IUD use with specific metabolic patterns are detectable after discontinuation of its use, additional linear regression models (adjusted for age, BMI, season of sampling, alcohol use, smoking, and physical activity) compared the associations of current and previous use of LNG-IUDs and all the metabolic measures with never-users of LNG-IUD as the reference category. Because information on past use of LNG-IUD was not available in the Health 2011, these analyses were performed in the Health 2000 dataset only.

For the longitudinal part of the study, the 240 users of LNG-IUD and non-users of any HC in 2000 who also had available information on HC use (LNG-IUD vs no HC) and metabolomics data in 2011 were selected. Four groups were identified: (1) continuers, ie, women who used LNG-IUD at both time points; (2) stoppers, ie, women who used LNG-IUD in 2000 but no HC in 2011; (3) starters, ie, women who used no HC in 2000 but used LNG-IUD in 2011; and (4) never-users, who did not use any HC in 2000 or 2011. Changes in levels of each metabolite were computed as the difference between levels in 2011 and 2000. Linear regression models were further performed to compare changes in each metabolite level in the 4 groups of users of LNG-IUD, with never-users of HC being the reference category. Analyses were controlled for age at baseline and change in BMI.

To take the multiple testing into account, we applied the false discovery ratio procedure, which considers the expected proportion of false discoveries among the rejected hypotheses. All the analyses were performed with R software version 4.2.3 (24).

## Results

Table 1 reports the baseline characteristics of the 1767 participants with available information on their current use of LNG-IUD and metabolomics data. A total of 341 individuals (19.3%) were using a LNG-IUD at the time of the surveys; they were older than those not using any HC (mean ± SD age 41.5 ± 6.1 vs 39.5 ± 5.1 years) and more likely to be employed (88.0% vs 76.1%), to be parous (97.9% vs 86.2%), and to use alcohol (96.2% vs 90.1%).

**Figure 1.**
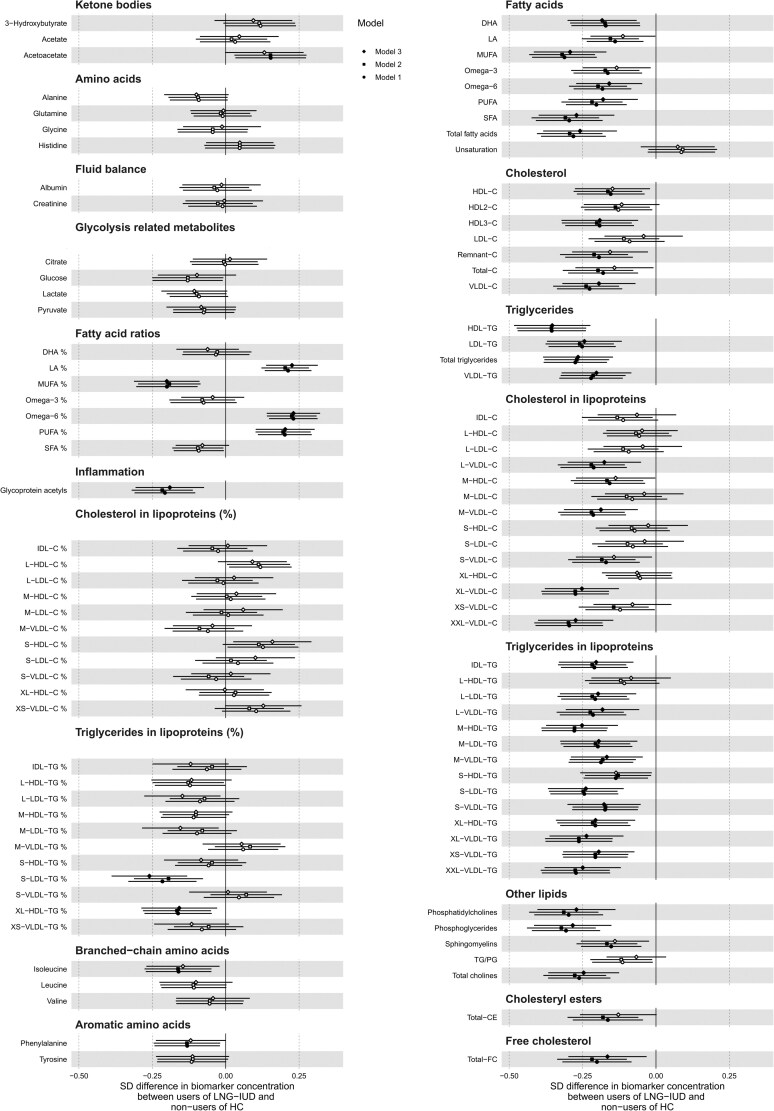

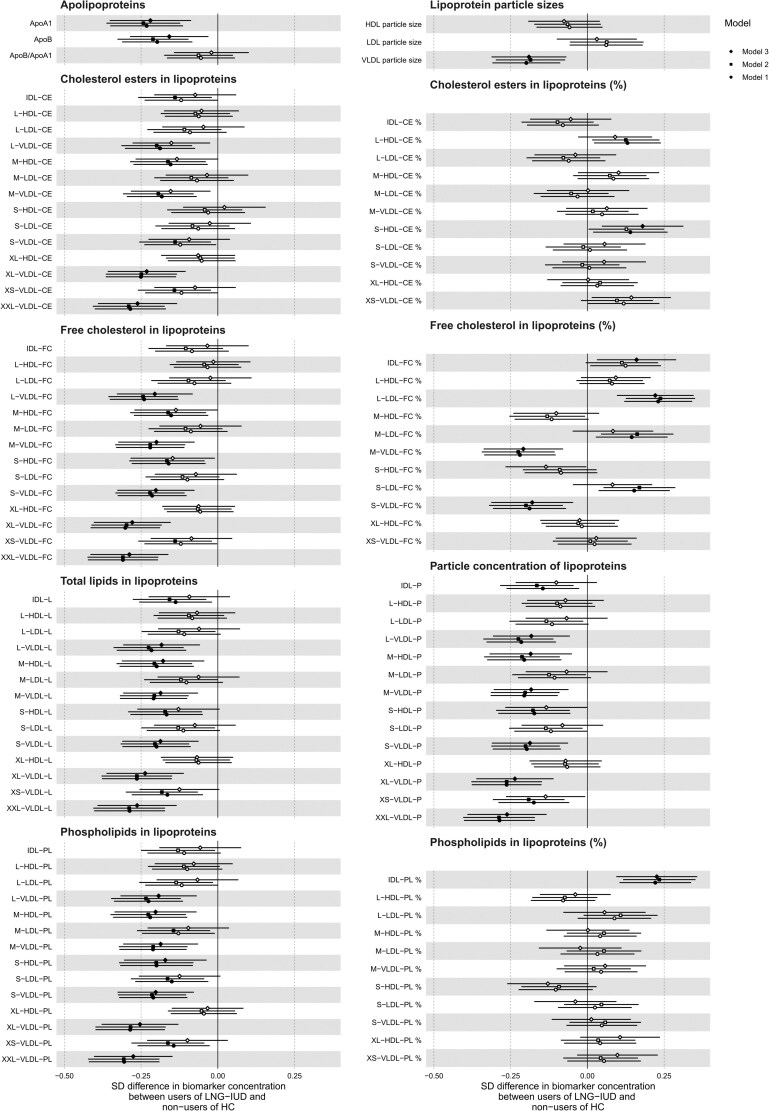
Associations between the use of a levonorgestrel-releasing intrauterine device and 212 metabolic measures. Analyses conducted in pooled Health 2000 and Health 2011 surveys. Model 1 is adjusted by age, body mass index, season of sampling, and study cohort; model 2 is model 1 further adjusted for disease and medication use; model 3 is model 1 further adjusted for lifestyle habits (alcohol use, smoking, and physical activity). Results are in SD units of difference in metabolite concentrations; bars indicate 95% confidence interval; reference category is non-users of hormonal contraceptives. Closed circles indicate significant associations at *P*-value adjusted for false discovery rate.

**Figure 2.**
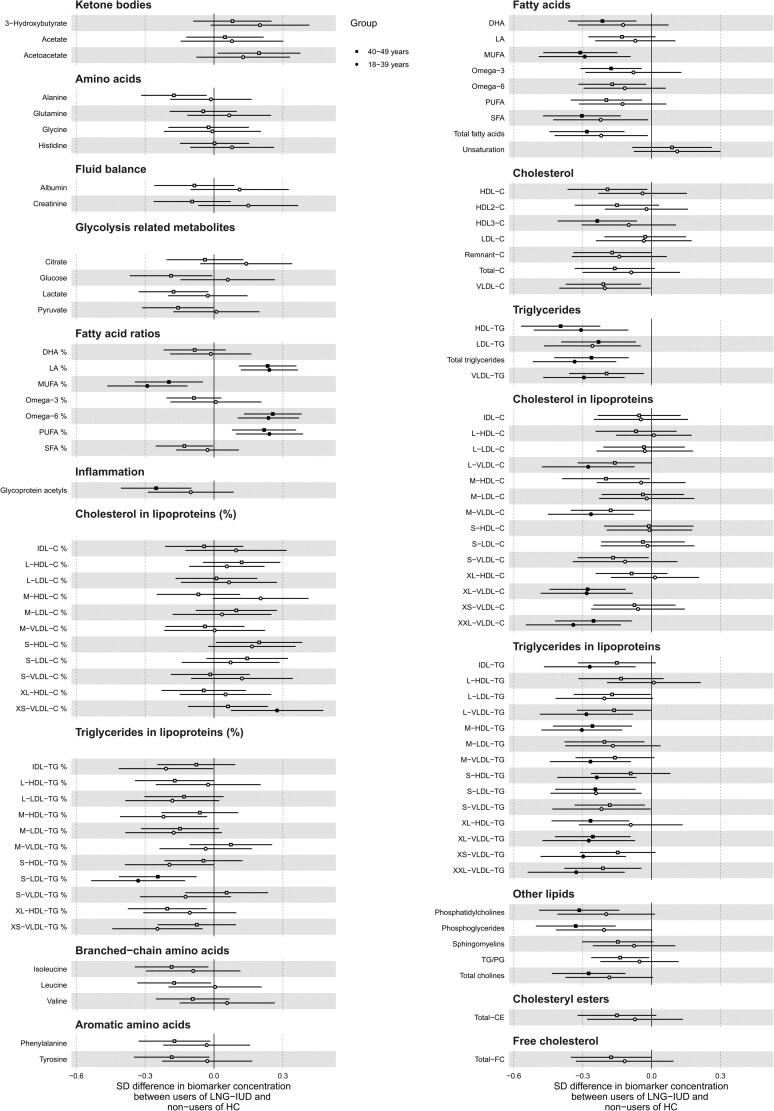

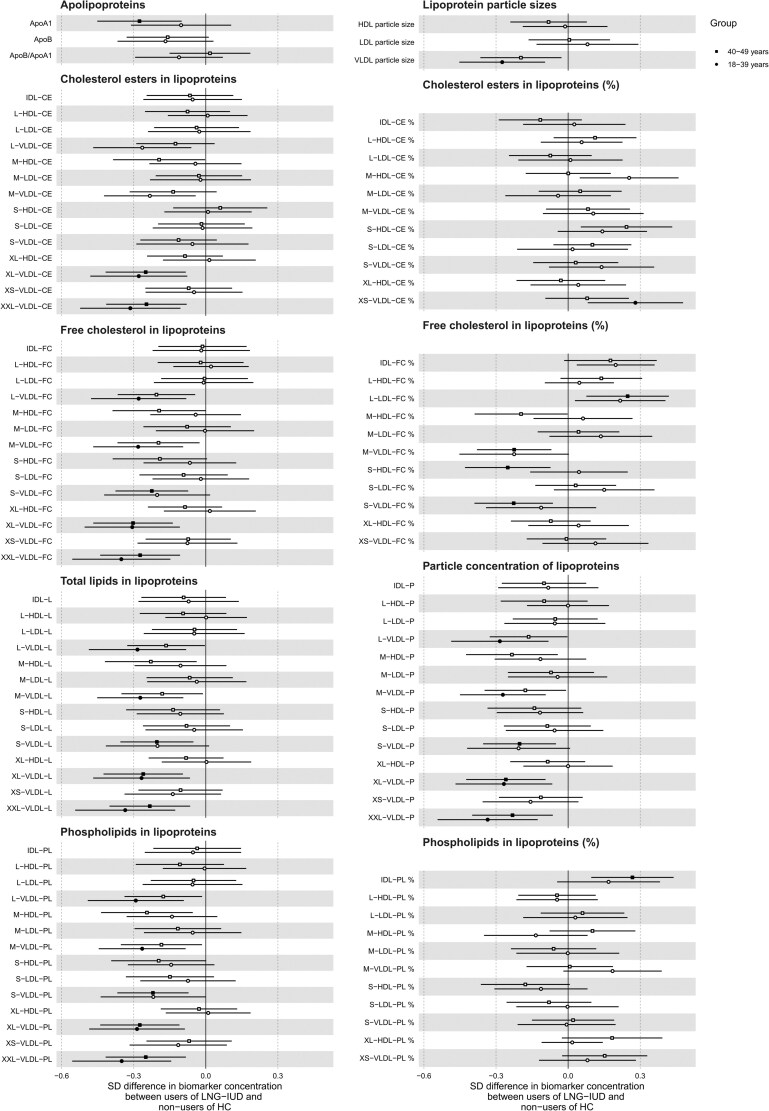
Age-stratified associations between the use of a levonorgestrel-releasing intrauterine device and 212 metabolic measures. Analyses conducted in pooled Health 2000 and Health 2011 surveys. Analyses are adjusted by age, body mass index, season of sampling, study cohort, alcohol use, smoking, and physical activity. Results are in SD units of difference in metabolite concentrations; bars indicate 95% confidence interval; reference category is non-users of hormonal contraceptives. Closed circles indicate significant associations at *P*-value adjusted for false discovery rate.

In linear regression analyses adjusted for age, BMI, season of sampling, and study cohort (model 1), 102 out of the 212 metabolic measures differed between LNG-IUD users and non-users of HC (median difference in biomarker concentration: −0.12 SD), in particular fatty acids concentrations and ratios, cholesterol, triglycerides, and other lipids, as well as particle concentration, cholesterol, triglycerides, total lipids, and phospholipids in lipoproteins (Fig. 1). The pattern of associations remained substantially unchanged after adjustment for disease and medication use (model 2); adjustment for lifestyle habits mitigated the associations with cholesterol and other lipids, while the associations of triglycerides and fatty acids remained mostly unchanged (model 3) (Fig. 1). Analyses conducted with GEE models to take correlation of observations of the same individual into account provided similar results (data not shown). In GEE, point estimates remain the same, but standard errors are increased. The results did not change after exclusion of women younger than 30 years of age (n = 47) (Supplementary Fig. S6) (18). The patterns of associations varied in different age groups: in particular, associations with lower levels of inflammation marker, fatty acids, and other lipids were mostly driven by the oldest age group (40-49 years, n = 972), while associations with lower levels of particle concentration, and in detail of cholesterol, triglycerides, free cholesterol, total lipids, and phospholipids in lipoproteins, were mostly evident in younger women (18-39 years, n = 795) (Fig. 2).

**Figure 3.**
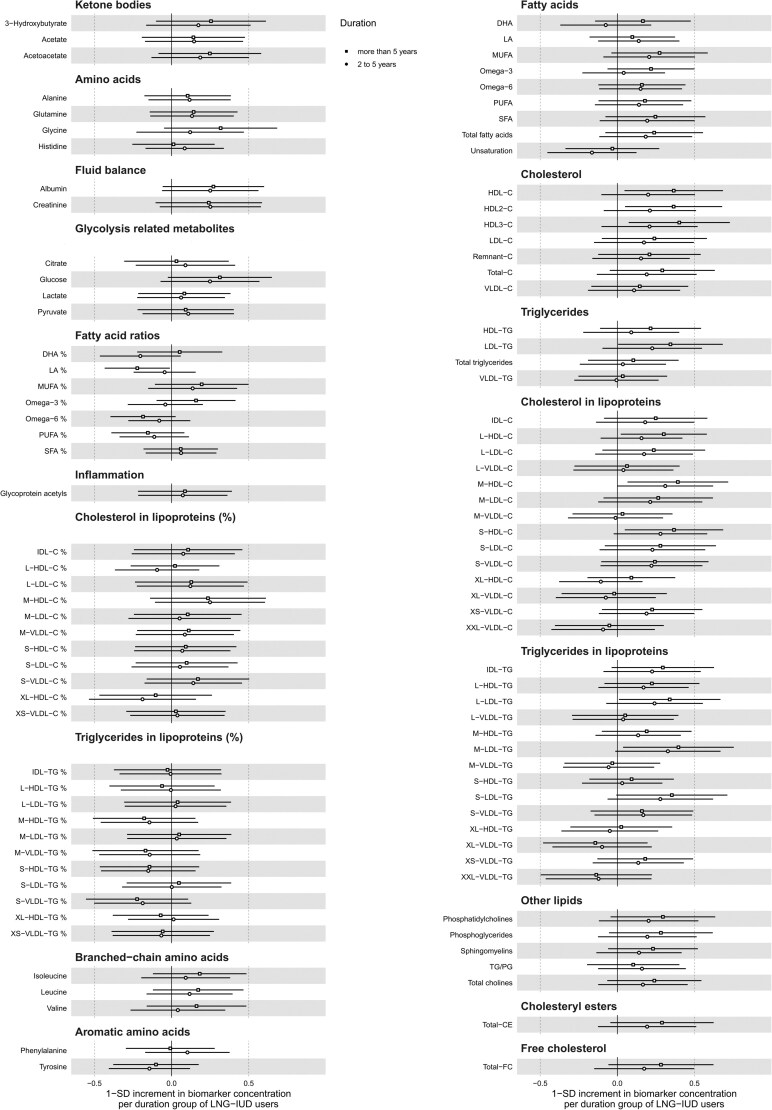

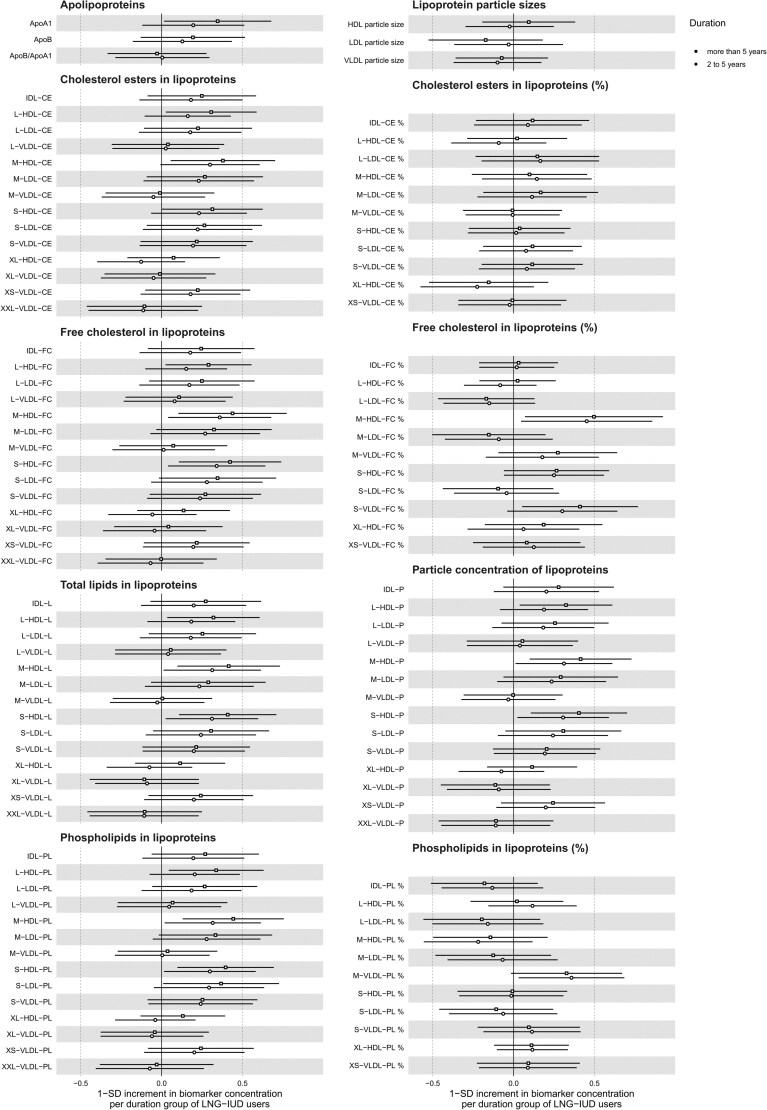
Associations between duration of use of a levonorgestrel-releasing intrauterine device and 212 metabolic measures. Analyses conducted in the group of current hormonal intrauterine device users only in the pooled Health 2000 and Health 2011 surveys. Analyses are adjusted by age, body mass index, season of sampling, study cohort, alcohol use, smoking, and physical activity. Results are in SD units of differences in metabolite concentrations; bars indicate 95% confidence interval; reference category is short-term (up to 2 years) use of a hormonal intrauterine device. Closed circles indicate significant associations at *P*-value adjusted for false discovery rate.

To examine the impact of duration of use on the associations between the use of LNG-IUD and metabolic profiles, we performed regression analyses in the subgroup of current users only, comparing the metabolic profile of intermediate-term (n = 129) and long-term (n = 154) use with short-term use (n = 58). No significant differences were found between the 3 groups (Fig. 3).

We further compared the metabolic profiles of current (n = 168) and previous users (n = 85) of LNG-IUDs with those of never-users (n = 938) in the Health 2000 Survey (Supplementary Table S2) (18). A model adjusted for age, BMI, season of sampling, alcohol use, smoking, and physical activity did not show any significantly different profiles in current or previous users compared to never-users of LNG-IUD (Supplementary Fig. S7) (18).

**Figure 4.**
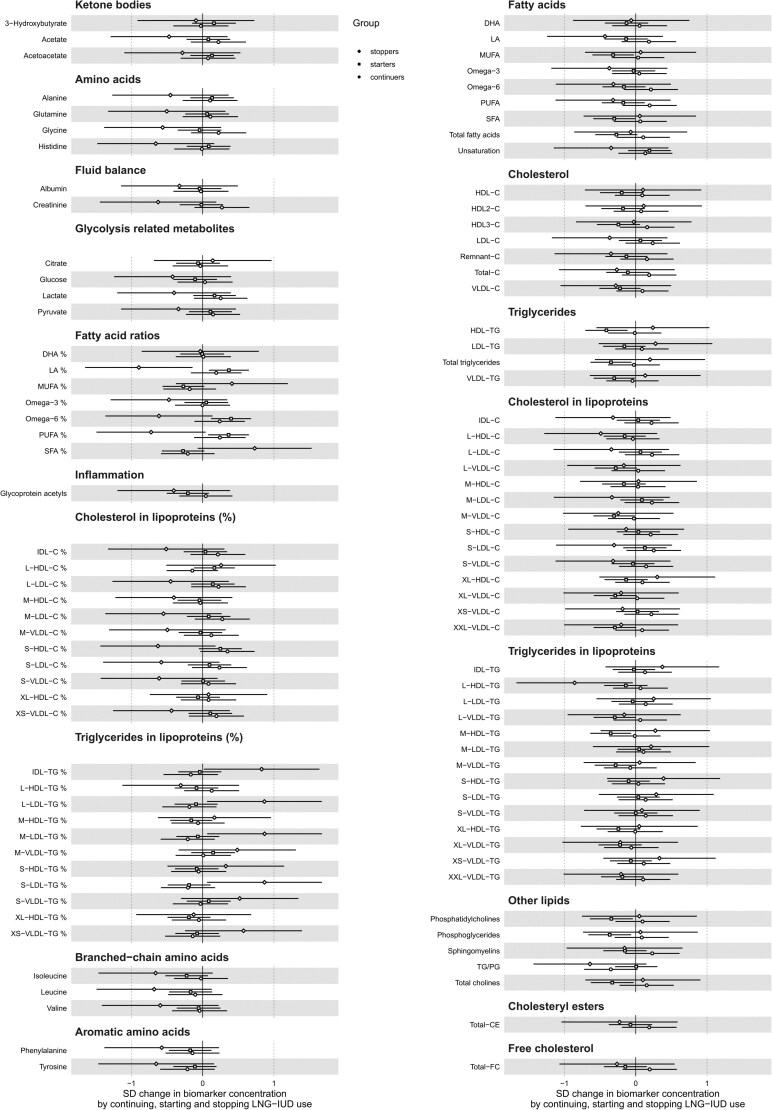

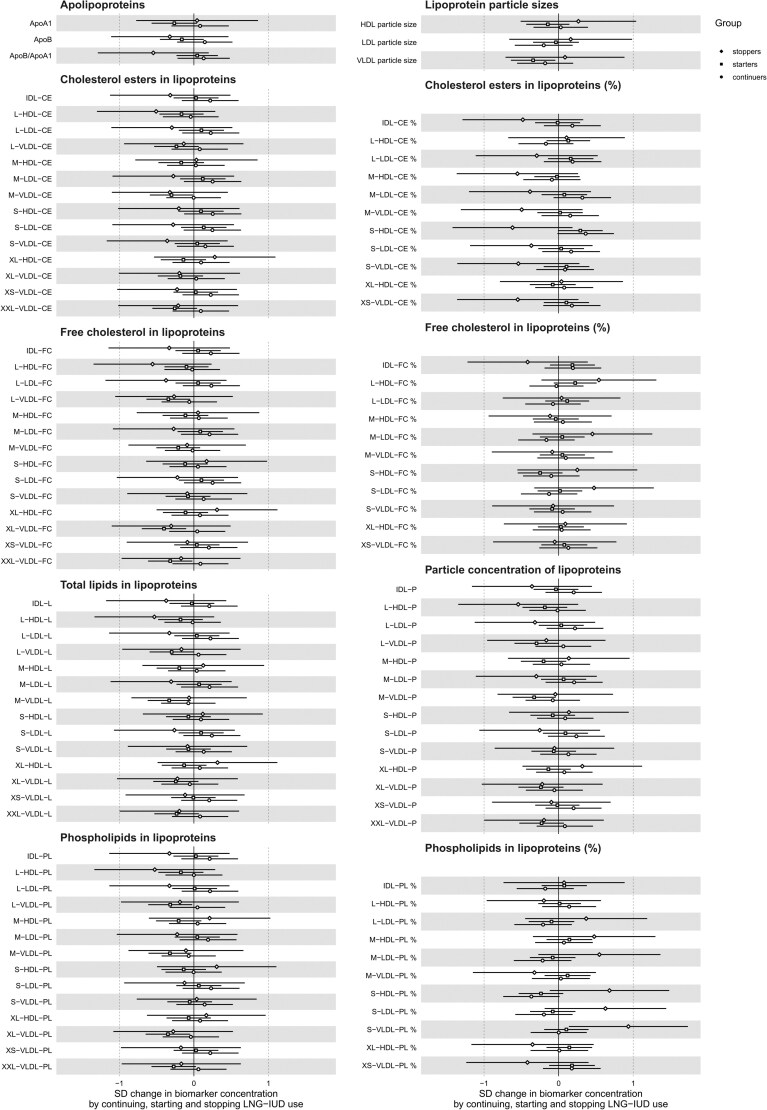
Eleven-year changes in molecular concentrations of 212 metabolic measures by starting, continuing, and stopping use of a levonorgestrel-releasing intrauterine device. Analyses are adjusted by age at baseline and 11-year change in body mass index. Results are in SD units of change in metabolite concentrations; bars indicate 95% confidence interval; reference category is never-users of hormonal contraception. Closed circles indicate significant associations at *P*-value adjusted for false discovery rate.

In the longitudinal analyses of the 11-year change in metabolic profiles of starters (n = 59), continuers (n = 33), and stoppers (n = 6) of LNG-IUD compared to never-users of HC (n = 142) (Table 2), no significant differences were found (Fig. 4).

**Table 2.** Background characteristics of the 240 participants included in the longitudinal study

	Starters n = 59	Stoppers n = 6	Continuers n = 33	Never users n = 142
Baseline age	33.9 (2.1)	34.5 (3.3)	35.2 (2.5)	33.9 (2.5)
BMI baseline	24.0 (4.1)	27.5 (5.6)	24.1 (3.9)	24.4 (4.2)
BMI follow-up	25.4 (4.1)	29.9 (8.5)	25.7 (4.7)	26.3 (4.5)
Alcohol use baseline	59 (100)	5 (83.3)	31 (93.9)	129 (91.5)
Alcohol use follow-up	58 (98.3)	6 (100)	31 (96.9)	124 (87.9)
Smoking baseline (%)				
Every day	12 (20.3)	2 (33.3)	5 (15.2)	31 (21.8)
Sometimes	1 (1.7)	0	3 (9.1)	11 (7.7)
No	46 (78.0)	4 (66.7)	25 (75.8)	100 (70.4)
Smoking follow-up (%)				
Every day	9 (15.3)	1 (16.7)	4 (12.1)	22 (15.5)
Sometimes	1 (1.7)	1 (16.7)	0	8 (5.6)
No	49 (83.1)	4 (66.7)	29 (87.9)	112 (78.9)
Physical activity baseline (%)				
Hardly any regular weekly physical activity	44 (74.6)	6 (100)	25 (75.8)	104 (73.8)
Physical activity yes	15 (25.4)	0	8 (24.2)	37 (26.2)
Physical activity follow-up (%)				
Hardly any regular weekly physical activity	32 (84.2)	1 (50.0)	18 (94.7)	75 (88.2)
Physical activity yes	6 (15.8)	1 (50.0)	1 (5.3)	10 (11.8)
Current use (past week) of any medication baseline*^a^* (%)	18 (32.1)	2 (33.3)	13 (39.4)	34 (25.4)
Current use (past week) of any medication follow-up*^a^* (%)	28 (47.5)	3 (50.0)	14 (42.4)	60 (42.3)
Chronic disease (ever or past year) baseline*^b^* (%)	11 (18.6)	2 (33.3)	6 (18.2)	49 (34.5)
Chronic disease (ever or past year) follow-up*^b^* (%)	18 (30.5)	3 (50.0)	12 (36.4)	59 (41.5)

Abbreviation: BMI, body mass index.

^*a*^Insulin, oral hypoglycemics, antithrombotic therapy, lipid medications, combination products of estrogens and progestins, estrogens, progestogens, systemic corticosteroids, systemic antibiotics, systemic antimycotics, cytostatics, anti-inflammatory analgesics, opioids, other analgesics (no nonsteroidal anti-inflammatory drug group), migraine medications, epilepsy medications, antipsychotics, anxiolytics, hypnotics and sedatives, antidepressants, psycholeptics and psychoanaleptics in combination, long- and short-acting beta agonists, combination product of beta agonist and corticosteroids, inhaled corticosteroids, anticholinergics.

^*b*^Heart diseases, hypertension, venous thrombosis, diabetes, psychological or mental illnesses, rheumatoid arthritis, osteoarthritis, cancer.

## Discussion

The main finding of this study is that the metabolic profile of current users of LNG-IUD differed in a relatively large number of metabolites compared to that of non-users of HC, but the magnitude of the associations was mostly from low to moderate. The detected associations were indicative of a reduced arterial cardiovascular risk, with lower levels (compared to non-users of HC) of fatty acids concentrations and ratios, triglycerides and other lipids, total lipids, and phospholipids in lipoproteins. The metabolic profile appeared not to vary with the duration of use, nor did we find any significant metabolic pattern related to 11-year changes in LNG-IUD use.

The overall results of this study are in line with our previous study (15), showing that the current use of LNG-IUD might be related to benign metabolic alterations. However, the magnitude of these alterations is likely to be of marginal importance. In the current study, the median difference in biomarker concentration between the users of LNG-IUD and non-users of HC was −0.12 SD. In line with the results of our previous study based on larger groups, we did not observe any difference in relation to the duration of use of LNG-IUD. In age-stratified analyses, this pattern of associations with lower levels of cholesterol, triglycerides, free cholesterol, total lipids, and phospholipids in lipoproteins was mostly evident in younger women. Although possibly explained by a selection bias, with women who were wealthier, more health-conscious, and in a long-term relationship being more likely to purchase LNG-IUD, altogether, these observations likely reflect the ongoing trend for LNG-IUD to be used at an increasingly younger age in Finland.

To the best of our knowledge, only 1 previous study has examined the metabolic effects of LNG-IUD in a longitudinal pattern, and it found no metabolic differences between starters, stoppers, and persistent users of LNG-IUD compared to persistent non-users (14). Similarly, in our study we found no metabolic changes related to changes in the use of LNG-IUD. Given the cross-sectional associations described here, the lack of a significant impact of starting or stopping LNG-IUD is somehow counterintuitive, although in line with the study by Wang et al (14). As such, it is likely that the null finding of our study, as well as that of Wang et al (14), is attributable to a lack of power consequent to the small subgroup sizes. However, because the detected cross-sectional associations are in the direction of protective effects, longitudinal associations in a larger sample, if any, were expected to be in the same direction. Taken together, the cross-sectional protective associations and the null finding in the longitudinal study point to a relative metabolic safety of LNG-IUD use.

As in our previous work, the majority of the associations were related to the absolute rather than relative concentrations of lipids and lipids in lipoproteins (Fig. 1). In detail, absolute levels of cholesterol and triglycerides in lipoproteins were especially lower (compared to non-users) in the XL- and XXL-very-low-density lipoproteins, and to a lesser extent, in the LDL and intermediate-density lipoproteins subclasses, which altogether carry the highest risk for myocardial infarction (25) and other cardiovascular events (26, 27). Similarly, we observed a general decrease in absolute levels of all fatty acids. On the other hand, the results for fatty acid proportions of the total fatty acids were more difficult to interpret. Fatty acids ratios are biological meaningful measures that better reflect the biology of individual fatty acids than the absolute concentrations and have proven distinct population distributions (19, 20). In our study, we found a lower ratio of monounsaturated fatty acids but higher ratio of omega 6, linoleic acid, and polyunsaturated fatty acids (PUFA) in users of LNG-IUD (suggesting relative higher proportion of omega 6 than omega 3 within PUFA). This observation is rather counterintuitive, as both reduced and increased cardiovascular risk (mostly explained by its proinflammatory effect) has been shown in relation to omega 6 PUFA and its precursor linoleic acid (28, 29). On the other hand, high levels of omega 3 have a clearer protective role in terms of cardiovascular risk (30), while effects of monounsaturated fatty acids are less clear (31).

Although we observed reduced levels of total HDL cholesterol associated with the use of LNG-IUD, we were able to obtain a detailed profile of HDL subfractions by means of metabolomics. In contrast to our previous work, where HDL2 appeared slightly more reduced than smaller and denser HDL3, we currently found lower levels of HDL3 but not total HDL or HDL2 in users of hormonal IUDs. A reason for these different findings could be the possible inclusion of users of 13.5 mg and 19.5 mg LNG-IUDs, along with the much larger sample size, in our previous study. As low levels of HDL2 are related to dyslipidemia and high levels of HDL3 to an increased risk of coronary heart disease (32), the current findings further support the potentially beneficial effects of LNG-IUD.

Finally, the protective metabolic profile of LNG-IUD users is further confirmed by the additional finding of reduced levels of glycoprotein acetyls, an inflammation marker that has emerged as a predictor for diabetes, cardiovascular diseases, and all-cause mortality (33), in users of a LNG-IUD, as also previously shown in our study on hormonal IUD (15).

The lack of differences between current, previous, and never users of LNG-IUD is opposite to findings of our previous study, conducted in a larger population, showing a consistently different profile in current vs never users of LNG-IUD. As the analyses were performed in a smaller sample inclusive of only Health 2000 data, it cannot be excluded that our current finding is a false negative due to the small size of the 3 subgroups. However, we expect that, if differences were to be found, they would have been in the same direction as the main findings and as such be suggestive of a nonharmful metabolic profile related to LNG-IUD use.

Taken together, our findings support the notion of the relative safety of LNG-IUD in terms of metabolic effects. Not only did the use of LNG-IUD, either short term or long term, appear to not carry an increased risk of adverse metabolic profiles, but it seemed to be related to a potentially cardioprotective pattern. These results confirm previous evidence, indicating no difference in serum concentrations of total and LDL cholesterol, triglycerides, insulin, glucose, and C-reactive protein (34, 35), or a reduction of total cholesterol, APO-A1, and APO-B (34, 36) in relation to LNG-IUD use. Additionally, findings from a randomized controlled trial (36) and a population-based cross-sectional study (37) indicate that short-term LNG-IUD use is associated with a decrease of HDL cholesterol, while long-term use is related to unchanged or increased levels of HDL cholesterol. In future longitudinal studies, the relation of LNG-IUD use and its protective metabolic profile to long-term clinical cardiometabolic events, as well as whether there are long-term beneficial metabolic effects of LNG-IUD use in menopause, should be further addressed.

### Strengths and Limitations

This study has some limitations. The first is related to the small sample size especially in subgroup and longitudinal analyses. Thus, it is likely that the absence of differences in subgroup analyses is due to a lack of statistical power. However, the null findings, eg, in relation to the duration of use of LNG-IUD, are in line with the results of our previous study, conducted in a similar population but with larger groups, suggesting that it is likely to be a true finding. The generalizability of the findings is further limited, considering that the majority of users of LNG-IUD in our population were older than 30 years. Additionally, because only 52 mg LNG-IUD was available in Finland until 2013, our results cannot be generalized to users of low-dose LNG-IUDs (13.5 mg and 19.5 mg). However, for this same reason, our results are free from confounding due to different hormonal doses. Although we did not have complete information on conditions possibly affecting the type and use of contraception and the metabolic status, such as endometriosis and polycystic ovary syndrome, our results were controlled for covariates covering a large set of diseases and confounding conditions. Because of the likely high correlations between several metabolites, interpretation of the implications and importance of single metabolites may have been biased. The data of this study derived mostly from self-administered questionnaires, potentially introducing a recall bias. However, a self-report of contraception use has been shown reliable in specifically focused studies (38, 39). Additionally, reliability of the data was confirmed through consistency throughout multiple related questions. While our previous (15) and current study are similar in the basic aim of examining the relationship between the use of LNG-IUD and metabolic biomarkers and use the same metabolomics platform, the overall underlying methodology and design are different, as the Health 2000 and Health 2011 include a much more extensive physical examination and comprehensive interview and questionnaire-based data collection. Even though the current study population resulted in a smaller sample size compared to our previous work, the follow-up data allowed us to include a longitudinal design in the current analysis plan.

Strengths of the study include the large number of available metabolomics measures and the extensive information on possible confounders, such as lifestyle and health characteristics. Additionally, the utilized metabolomic platform provides a rather complete assessment of metabolic status, inclusive of absolute and relative metabolite measures with proven separate biological meaning (20, 21). The clinical significance of the different molecular ratios has been previously shown (19-21).

Further strength of the study is given by the availability of repeated measures for a subgroup of women, which allowed a longitudinal examination of the previously detected associations.

In spite of the relatively small sample size and consequently limited power, especially for subgroup and longitudinal analyses, there is a general lack of metabolomics studies on the topic, and the clinical studies that have to date evaluated metabolic outcomes of LNG-IUD are based on similar samples as ours. Additionally, it is not feasible to conduct randomized controlled trials comparing the effects of LNG-IUD vs non-use of HC on clinical cardiovascular outcomes. Thus, although our results have to be taken with caution, they represent important preliminary knowledge in the field that calls for future studies with larger samples.

## Conclusions

Our findings confirm previous observations on the use of LNG-IUD being associated with many multiple moderate metabolic changes, mostly suggestive of reduced arterial cardiometabolic risk. These associations appeared mostly to be independent of the duration of use, and changes in LNG-IUD use were not related to long-term metabolic changes. Taken together, our results support the relative metabolic safety of LNG-IUD.

## Data Availability

Restrictions apply to the availability of some or all data generated or analyzed during this study to preserve patient confidentiality or because they were used under license. The corresponding author will on request detail the restrictions and any conditions under which access to some data may be provided. The individual level data used in this manuscript is available through THL Biobank at https://thl.fi/en/web/thl-biobank/for-researchers.

## References

[1] Ahrens KA , Skjeldestad FE. Trends in initiation of hormonal contraceptive methods among teenagers born between 1989 and 1997 in Norway and the United States. Contraception. 2021;104(6):635‒641. 34329611 10.1016/j.contraception.2021.07.105

[2] Kristensen SI , Lidegaard Ø. Hormonal contraceptive use in Denmark 2010-2019. Dan Med J. 2021;68(6):A08200599. 34060463

[3] Jensen JT . Contraceptive and therapeutic effects of the levonorgestrel intrauterine system: an overview. Obstet Gynecol Surv. 2005;60(9):604‐612. 16121115 10.1097/01.ogx.0000175805.90122.af

[4] Mansour D . The benefits and risks of using a levonorgestrel-releasing intrauterine system for contraception. Contraception. 2012;85(3):224‐234. 22067761 10.1016/j.contraception.2011.08.003

[5] Aleknaviciute J , Tulen JHM, De Rijke YB, et al The levonorgestrel-releasing intrauterine device potentiates stress reactivity. Psychoneuroendocrinology. 2017;80:39‐45. 28315609 10.1016/j.psyneuen.2017.02.025

[6] Lopez LM , Chen RS, Edelman M, et al Progestin-only contraceptives: effects on weight. Cochrane Database Syst Rev. 2016;8:CD008815. 10.1002/14651858.CD008815.pub4 PMC5034734 27567593

[7] Heikinheimo O , Toffol E, Partonen T, But A, Latvala A, Haukka J. Systemic hormonal contraception and risk of venous thromboembolism. Acta Obstet Gynecol Scand. 2022;101(8):846‐855. 35633036 10.1111/aogs.14384 PMC9564731

[8] Rosano GMC , Rodriguez-Martinez MA, Spoletini I, Regidor PA. Obesity and contraceptive use: impact on cardiovascular risk. ESC Heart Fail. 2022;9(6):3761‐3767. 36103980 10.1002/ehf2.14104 PMC9773763

[9] Dorflinger LJ . Metabolic effects of implantable steroid contraceptives for women. Contraception. 2002;65(1):47‐62. 11861055 10.1016/s0010-7824(01)00290-6

[10] Dal'Ava N , Bahamondes L, Bahamondes MV, de Oliveira Santos A, Monteiro I. Body weight and composition in users of levonorgestrel-releasing intrauterine system. Contraception. 2012;86(4):350‐353. 22445431 10.1016/j.contraception.2012.01.017

[11] Modesto W , de N, Silva dos Santos P, Correia VM, Borges L, Bahamondes L. Weight variation in users of depot-medroxyprogesterone acetate, the levonorgestrel-releasing intrauterine system and a copper intrauterine device for up to ten years of use. Eur J Contracept Reprod Health Care. 2015;20(1):57‐63. 25160484 10.3109/13625187.2014.951433

[12] Glisic M , Shahzad S, Tsoli S, et al Association between progestin-only contraceptive use and cardiometabolic outcomes: a systematic review and meta-analysis. Eur J Prev Cardiol. 2018;25(10):1042‐1052. 29745237 10.1177/2047487318774847 PMC6039863

[13] Kalenga CZ , Dumanski SM, Metcalfe A, et al The effect of non-oral hormonal contraceptives on hypertension and blood pressure: a systematic review and meta-analysis. Physiol Rep. 2022;10(9):e15267. 35510324 10.14814/phy2.15267 PMC9069167

[14] Wang Q , Würtz P, Auro K, et al Effects of hormonal contraception on systemic metabolism: cross-sectional and longitudinal evidence. Int J Epidemiol. 2016;45(5):1445‐1457. 27538888 10.1093/ije/dyw147 PMC5100613

[15] Toffol E , Heikinheimo O, Jousilahti P, et al Metabolomics profile of 5649 users and nonusers of hormonal intrauterine devices in Finland. Am J Obstet Gynecol. 2022;227(4):603.e1‐603.e29. 10.1016/j.ajog.2022.06.009 35697093

[16] Heistaro S (ed.). Methodology Report: Health 2000 Survey. Kansanterveyslaitoksen julkaisuja B26/2008; 2008.

[17] Lundqvist A , Mäki-Opas T, eds. Health 2011 Survey - Methods. Terveyden ja hyvinvoinnin laitos, Raportti 8/2016; 2016:1‐219.

[18] Toffol E , Heikinheimo O, Jousilahti P, et al Supplementary Figures: “Moderate Associations between the use of levonorgestrel-releasing intrauterine device and metabolomics profile”. *Zenodo*. 2024. Deposited 9 May 2024. 10.5281/zenodo.11163800 PMC11913106 38717898

[19] Soininen P , Kangas AJ, Würtz P, Suna T, Ala-Korpela M. Quantitative serum nuclear magnetic resonance metabolomics in cardiovascular epidemiology and genetics. Circ Cardiovasc Genet. 2015;8(1):192‐206. 25691689 10.1161/CIRCGENETICS.114.000216

[20] Würtz P , Kangas AJ, Soininen P, Lawlor DA, Davey Smith G, Ala-Korpela M. Quantitative serum nuclear magnetic resonance metabolomics in large-scale epidemiology: a primer on -omic technologies. Am J Epidemiol. 2017;186(9):1084‐1096. 29106475 10.1093/aje/kwx016 PMC5860146

[21] Wang J , Stančáková A, Soininen P, et al Lipoprotein subclass profiles in individuals with varying degrees of glucose tolerance: a population-based study of 9399 Finnish men. J Intern Med. 2012;272(6):562‐572. 22650159 10.1111/j.1365-2796.2012.02562.x

[22] Liaw A , Wiener M. Classification and regression by randomForest. R News. 2002;2:18‐22.

[23] Scheinin I , Kalimeri M, Jagerroos V, et al *Forestplots of Measures of Effects and Their Confidence Intervals*. 2023. Accessed May 13, 2024. https://nightingalehealth.github.io/ggforestplot/index

[24] R Core Team . R: A Language and Environment for Statistical Computing. R Foundation for Statistical Computing; 2023. URL https://www.R-project.org/

[25] Balling M , Afzal S, Varbo A, et al VLDL cholesterol accounts for one-half of the risk of myocardial infarction associated with apoB-containing lipoproteins. J Am Coll Cardiol. 2020;76(23):2725‐2735. 33272366 10.1016/j.jacc.2020.09.610

[26] Norata GD , Raselli S, Grigore L, et al Small dense LDL and VLDL predict common carotid artery IMT and elicit an inflammatory response in peripheral blood mononuclear and endothelial cells. Atherosclerosis. 2009;206(2):556‐562. 19376517 10.1016/j.atherosclerosis.2009.03.017

[27] Ito K , Yoshida H, Yanai H, et al Relevance of intermediate-density lipoprotein cholesterol to Framingham risk score of coronary heart disease in middle-aged men with increased non-HDL cholesterol. Int J Cardiol. 2013;168(4):3853‐3858. 23850319 10.1016/j.ijcard.2013.06.023

[28] Maki KC , Eren F, Cassens ME, Dicklin MR, Davidson MH. ω-6 polyunsaturated fatty acids and cardiometabolic health: current evidence, controversies, and research gaps. Adv Nutr. 2018;9(6):688‐700. 30184091 10.1093/advances/nmy038 PMC6247292

[29] Davinelli S , Intrieri M, Corbi G, Scapagnini G. Metabolic indices of polyunsaturated fatty acids: current evidence, research controversies, and clinical utility. Crit Rev Food Sci Nutr. 2021;61(2):259‐274. 32056443 10.1080/10408398.2020.1724871

[30] Skeaff CM , Miller J. 2009. Dietary fat and coronary heart disease: summary of evidence from prospective cohort and randomised controlled trials. Ann Nutr Metab. 2009; 55(1-3):173‐201. 19752542 10.1159/000229002

[31] Skidmore PM , Woodside JV, Mc Master C, et al Plasma free fatty acid patterns and their relationship with CVD risk in a male middle-aged population. Eur J Clin Nutr. 2010;64(3):239‐244. 20087373 10.1038/ejcn.2009.144

[32] Pirillo A , Norata GD, Catapano AL. High-density lipoprotein subfractions–what the clinicians need to know. Cardiology. 2013;124(2):116‐125. 23428644 10.1159/000346463

[33] Lawler PR , Akinkuolie AO, Chandler PD, et al Circulating N-linked glycoprotein acetyls and longitudinal mortality risk. Circ Res. 2016;118(7):1106‐1115. 26951635 10.1161/CIRCRESAHA.115.308078 PMC4836171

[34] Nilsson CG , Kostiainen E, Ehnholm C. Serum lipids and high-density-lipoprotein cholesterol in women on long-term sustained low-dose IUD treatment with levonorgestrel. Int J Fertil. 1981;26(2):135‐137. 6114067

[35] Morin-Papunen L , Martikainen H, McCarthy MI, et al Comparison of metabolic and inflammatory outcomes in women who used oral contraceptives and the levonorgestrel-releasing intrauterine device in a general population. Am J Obstet Gynecol. 2008;199(5):529.e1‐529.e10. 10.1016/j.ajog.2008.04.013 18533124

[36] Ng YW , Liang S, Singh K. Effects of Mirena (levonorgestrel-releasing intrauterine system) and Ortho Gynae T380 intrauterine copper device on lipid metabolism–a randomized comparative study. Contraception. 2009;79(1):24‐28. 19041437 10.1016/j.contraception.2008.07.012

[37] Graff-Iversen S , Tonstad S. Use of progestogen-only contraceptives/medications and lipid parameters in women age 40 to 42 years: results of a population-based cross-sectional Norwegian survey. Contraception. 2002;6681(1):7‐13. 10.1016/s0010-7824(02)00311-6 12169374

[38] Sieving R , Hellerstedt W, McNeely C, Fee R, Snyder J, Resnick M. Reliability of self-reported contraceptive use and sexual behaviors among adolescent girls. J Sex Res. 2005;42(2):159‐166. 16123846 10.1080/00224490509552269

[39] Smith C , Edwards P, Free C. Assessing the validity and reliability of self-report data on contraception use in the MObile technology for improved family planning (MOTIF) randomised controlled trial. Reprod Health. 2018;15(1):50. 29544520 10.1186/s12978-018-0494-7 PMC5856309

